# Abnormal eosinophils with immature eosinophilic granules in chronic myeloid leukemia in accelerated phase

**DOI:** 10.1002/ccr3.4783

**Published:** 2021-09-07

**Authors:** Jennifer Cai, Ping Ji, Sarah Tomassetti, Xin Qing

**Affiliations:** ^1^ Department of Pathology Harbor‐UCLA Medical Center Torrance California USA; ^2^ Department of Internal Medicine Harbor‐UCLA Medical Center Torrance California USA

**Keywords:** abnormal eosinophils, accelerated phase, acute myeloid leukemia with inv(16)(p13.1q22) or t(16;16)(p13.1;q22), CBFB‐MYH11, chronic myeloid leukemia

## Abstract

Abnormal eosinophils with immature eosinophilic granules are typically observed in acute myeloid leukemia with inv (16) (p13.1q22) or t (16;16) (p13.1;q22) but can also be seen in chronic myeloid leukemia without inv (16) or t (16;16).

## Graphical abstract

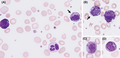

## 1 CASE

Abnormal eosinophils with immature eosinophilic granules are the usual morphological features seen in acute myeloid leukemia with inv (16) (p13.1q22) or t (16;16) (p13.1;q22). We report the presence of these cells in chronic myeloid leukemia in accelerated phase without inv (16) (p13.1q22) or t (16;16) (p13.1;q22).

**FIGURE 1:**
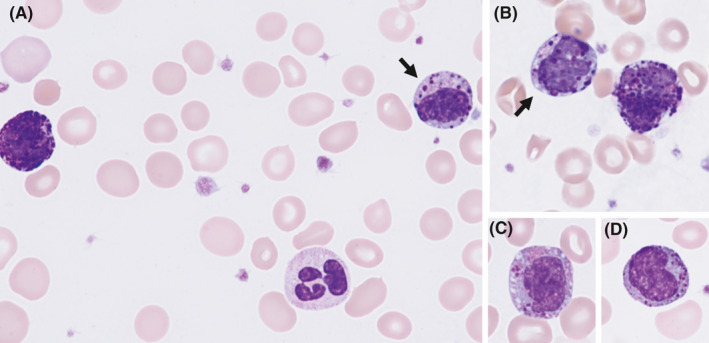
Peripheral blood smears showing abnormal eosinophils *[arrows in (A) and (B), (C), and (D)]* containing both eosinophilic granules and large “basophilic granules” (Wright‐Giemsa stain; original magnification×1000)

A 65‐year‐old woman with a history of chronic myeloid leukemia (CML) presented for follow‐up. She had been initially treated with imatinib, and she was then switched to dasatinib due to a suboptimal response. Subsequently, her therapy was switched to ponatinib due to the development of a T315I mutation. Ponatinib had been held for 1 month due to neutropenia. A complete blood count revealed anemia (hemoglobin, 8.7 g/dl), leukocytosis (54.8 × 10^9^/L), and thrombocytosis (601 × 10^9^/L). A peripheral blood smear showed 8% neutrophils, 1% myelocytes, 5% eosinophils, 4% lymphocytes, 81% basophils, and 1% blasts. Some of the eosinophils contain both eosinophilic granules and large basophilic colored granules (Figure 1). A bone marrow aspiration and biopsy demonstrated markedly hypercellular bone marrow with myeloid hyperplasia including many basophils and 2%–3% blasts. Chromosome analysis revealed t (9;22) and t (7;17) in all cells (46,XX,t [7;17] [p15;q23],t [9;22] [q34;q11.2] [20]). FISH analysis for *CBFB*‐*MYH11* was negative. Given the presence of ≥20% basophils in the peripheral blood, a diagnosis of CML in accelerated phase was rendered.

## 2 DISCUSSION

Abnormal eosinophils with immature eosinophilic granules are characteristically observed in acute myeloid leukemia with inv (16) (p13.1q22) or t (16;16) (p13.1;q22); *CBFB*‐*MYH11*. They have also been reported in CML with clonal evolution showing inv (16)/t (16;16) in addition to t (9;22).^1^, ^2^ The immature eosinophilic granules are often larger than those normally observed in immature eosinophils, are purple‐violet in color, and are most often found at the late promyelocyte and myelocyte stages. This case demonstrates that the presence of these cells does not always signify the presence of inv (16) (p13.1q22) or t (16;16) (p13.1;q22).

## CONFLICT OF INTEREST

None.

## AUTHOR CONTRIBUTIONS

JC wrote the article, prepared the figure, and did literature review. PJ and XQ edited the manuscript. ST discussed with the patient about the publication and obtained the written consent. XQ supervised this work. All authors have read and approved the final manuscript.

## ETHICAL APPROVAL

Ethical review and approval of the study are not applicable in this case.

## Data Availability

No datasets were generated or analyzed during the current study.
